# Localization and Characterization of Ferritin in Demospongiae: A Possible Role on Spiculogenesis

**DOI:** 10.3390/md12084659

**Published:** 2014-08-22

**Authors:** Filipe Natalio, Stefanie Wiese, Norman Friedrich, Peter Werner, Muhammad Nawaz Tahir

**Affiliations:** 1 Institut für Chemie—Anorganische Chemie, Naturwissenschaftliche Fakultät II—Chemie, Physik und Mathematik, Martin-Luther-Universität Halle-Wittenberg, Kurt-Mothes-Straße 2, Halle 06120, Germany; E-Mails: stefanie.wiese@chemie.uni-halle.de (S.W.); norman.friedrich@chemie.uni-halle.de (N.F.); 2 Max-Planck Institute of Microstructure Physics, Weinberg 2, Halle 06120, Germany; E-Mail: werner@mpi-halle.mpg.de; 3 Institut für Anorganische Chemie und Analytische Chemie, Johannes Gutenberg-Universität, Duesbergweg 10–14, Mainz 55099, Germany; E-Mail: tahir@uni-mainz.de

**Keywords:** *Suberites domuncula*, primmorphs, iron, ferritin, spiculogenesis

## Abstract

Iron, as inorganic ion or as oxide, is widely used by biological systems in a myriad of biological functions (e.g., enzymatic, gene activation and/or regulation). In particular, marine organisms containing silica structures—diatoms and sponges—grow preferentially in the presence of iron. Using primary sponge cell culture from *S. domuncula*–primmorphs—as an *in vitro* model to study the Demospongiae spiculogenesis, we found the presence of agglomerates 50 nm in diameter exclusively inside sponge specialized cells called sclerocytes. A clear phase/material separation is observed between the agglomerates and the initial stages of intracellular spicule formation. STEM-HRTEM-EDX analysis of the agglomerates (30–100 nm) showed that they are composed of pseudohexagonal nanoparticles between 5 and 15 nm in size, displaying lattice parameters corresponding to hematite (Fe_2_O_3_) and mixed iron oxide phases typically attributed to ferritin. Further analysis, using western blotting, inductively coupled plasma mass spectrometry (ICP-MS), sequence alignment analysis, immunostaining and magnetic resonance imaging (MRI), of mature spicule filaments confirm the presence of ferritin within these organic structures. We suggest that *S. domuncula* can be classified as a dual biomineralizating organism, *i.e.*, within the same cellular structure two distinct biomineralizing processes can occur as a result of the same cellular/metabolic function, spiculogenesis.

## 1. Introduction

Sponge fossil records show that these animals first appeared during the Neoproterozoic Era (1000 to 542 Ma) [1,2]. During this period, other multicellular animals also existed, which became extinct [3], especially during the Varanger-Marinoan ice ages (605 to 585 Ma). Neoproterozoic oceans (1000 to 542 Ma) were saturated with silicic acid and carbonates, and iron was continuously introduced by weathering cycles. Given their abundance, it can be easily inferred that these animals integrated silica and carbonate as fundamental elements for building-up of their inorganic skeleton [4]. 

In an exhaustive survey of biomineralizing animals, Lowenstam and Weiner elegantly proposed two concepts that distinguish two categories of mineralization: (i) biologically induced mineralization and (ii) biologically controlled mineralization, highlighting the fundamental importance of organic macromolecules during the biomineralization process [5]. As an example of the second concept, one can find corresponding skeletal elements in Hexactinellida and Demospongiae sponges. These two examples are composed of amorphous opal (SiO_2_•*n*H_2_O), and their biosilicification process is enzymatically controlled by a protein called silicatein (silica protein) [6,7,8]. Interestingly, it has been proposed that silicatein has a dual role, acting simultaneously as an organic framework and as a silica polymerizing enzyme [9,10].

Within the context of biologically controlled mineralization, the investigation of spiculogenesis in the model demosponge system *Suberites domuncula* is the most complete and exhaustive work completed until now on biosilicification processes *in vivo* [11]. The growth process of siliceous (SiO_2_) spicules has been elucidated using a 3D cell culture system from *S. domuncula*–primmorphs [12,13]. Recently, the use of transmission electron microscopy imaging on primmorph thin cuts showed that spicule formation in primmorphs starts intracellularly. In parallel, silicic acid is actively taken up by cells [sclerocytes] via the Na^+^/HCO_3_^−^[Si(OH)_4_] co-transporter [14] and deposited together with silicic acid in special organelles of the sclerocytes, the silicasome [15]. Mature silicatein is synthesized/processed in a parallel metabolic pathway. Their combination in a specialized vesicle allows the intracellular formation of spicules that further grow appositionally. It was recently suggested that the first appositional silica layers are deposited around a small crystalline aluminum silicate rod-like structure that acts as an axial filament precursor [16]. After reaching a certain size, the immature spicules are extruded from the cells into the extracellular space, where they proceed to grow in thickness (layer-by-layer) and in length [7,8,11].

Interestingly, primmorphs showed a significant increase in size when cultivated in a medium containing silicate with iron (30 μM) in comparison to only silicate (60 μM) [17]. It was also found that both silicatein and ferritin gene expression were simultaneously upregulated in the presence of silicate with iron (30 μM). Although the exact function of iron remains to be established, it plays a role in intracellular spiculogenesis.

Iron has a paradoxical relation in biology because it is a necessary element for life, but it also has the ability to form hazardous reactive oxygen species (ROS) [18]. Biomineralization of iron was developed by a wide range of evolutionarily distinct organisms, aside/apart from the various iron metabolic pathways [5]. Varying forms of (non-toxic) iron oxide biominerals can be found in different organisms, such as chitons [5] and magnetotactic bacteria [19], among others [20].

For example, magnetite (Fe_3_O_4_) is directly related to magneto-orientation function with the Earth’s magnetic field (e.g., *magnetotaxis*) [19]. Other iron oxide compounds such as lepidocrocite (γ-FeOOH) and ferrihydrite have been described, where the first mineralizes as unknown function granules within a sponge [21], and the latter is largely associated with the iron binding/storage protein called ferritin. From an evolutionary perspective, it can be assumed that ferritin has been developed by organisms to render intracellular iron inert [5]. The iron core of ferritin consists of ferrihydrite [22,23], an ubiquitous iron oxyhydroxide metastable mineral with the chemical formula Fe_5_HO_8_·4H_2_O [22,24].

This unique iron storage protein includes almost all the elements required for biomineralization: (i) an enzymatic catalysis for molecular transformation of precursor sites; (ii) a site for mineral nucleation and (iii) the architecture that defines and imposes physical limits on the final morphology of a biomineral. Ferritin is a spherical protein cage with an important function in regulating iron homeostasis and safely storing iron, sequestering and consequently encapsulating highly reactive, free Fe(II) ions (up to 4500 ions Fe(II) per structure), rendering them inactive and thereby minimizing potential cell damage [25]. Despite the fact that the primary amino acid sequences of ferritins show very low homology, the structural homology is highly conserved. All ferritins are composed of 24 structurally identical subunits that assemble into a proteinaceous cage with octahedral symmetry. The diameter of external and internal cavities is 12 and 6–8 nm, respectively. Mammalian ferritin is composed of two classes of subunits, structurally near-identical, named H-chain (heavy) and L-chain (light), which self-assemble to form hetero-24-mers with different H/L ratios, depending on the originating organism or organ [26].

Here we demonstrate the presence of ferritin and the typical iron core in marine sponge *S. domuncula* using primary sponge cell culture, primmorphs. For this purpose, the structural features of the inorganic core, found exclusively inside of sclerocytes, were determined by scanning transmission electron microscopy (STEM), high-resolution electron microscopy (HRTEM), and elemental analysis (EDX). The presence of ferritin was confirmed (i) in tissue extract by gel electrophoresis and western blotting techniques; (ii) in spicules and filaments by immunostaining techniques, ICP-MS and Magnetic Resonance Imaging (MRI); and (iii) in tissue sections by Perls’ Prussian Blue staining. Finally, we present a hypothetic role of iron in early stages of spiculogenesis.

## 2. Results and Discussion

### 2.1. Analysis of Intracellular Structures

**Figure 1:**
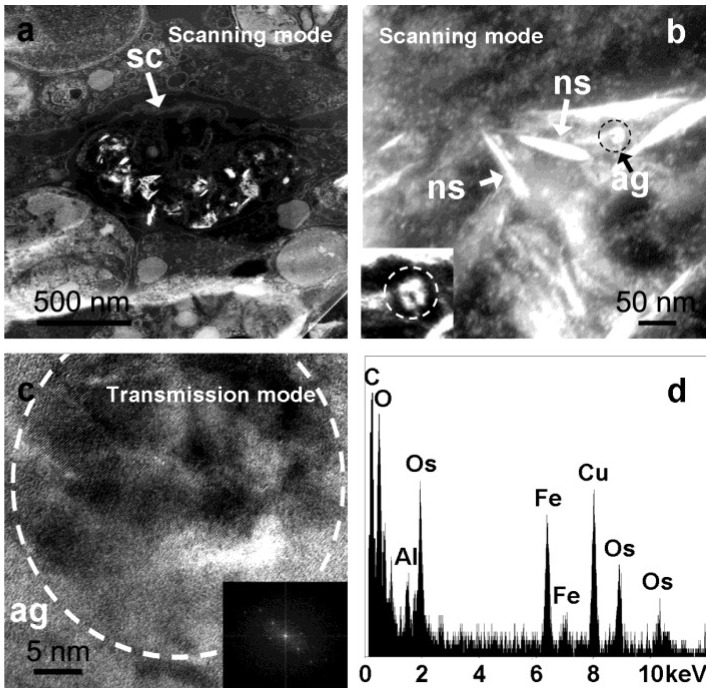
Sequence of magnifying electron microscope (EM) images of primmorphs sclerocytes’s ultrathin cuts under different modes. Scanning transmission electron microscope images (scanning mode) of (**a**) Overview of tissue containing sclerocyte (*sc*) filled with high electron-dense features; (**b**) The same sclerocyte at higher magnification showing the nanospicules (*ns*) and approximately 50 nm agglomerates (*ag—*indicated by the dashed black circle) and the corresponding magnification (inset); (**c**) High magnification (HRTEM) of scanning transmission electron microscope images (transmission mode) of agglomerates (*ag*—indicated by the dashed white circle); (**d**) Correspondent energy dispersive X-ray (EDX) spectrum shows the presence of mainly elements such as Fe, O, C, with traces of Al. Os is due to the sample methodology.

Primmorphs, sponge cell primary culture from *S. domuncula*, grown in artificial seawater (CMFASW) for 10 days were immobilized in an epoxy resin, and ultrathin cuts were analyzed using microscopic techniques as previously demonstrated [7,8,11,15,16]. Scanning transmission electron microscopy (STEM in scanning mode) analysis carried out on these ultrathin cuts of primmorphs showed a variety of features with different brightness inside specific sponge cells that are attributed to sclerocytes (Figure 1a). At a higher magnification of the same spot, scanning transmission electron microscopy (STEM in scanning mode—Figure 1b) images show electron-dense crystalline nanospicules (Figure 1b *ns*), which have been previously shown to be spicule templates [16]. Rounded, highly electron-dense features (agglomerates) of 50 nm in diameter can also be observed inside the sclerocytes (Figure 1b *ag* and inset). Performing high-resolution electron microscopy (STEM in transmission mode) of these nanosized agglomerates is not easy, as the surrounding organic material and the subsequent fast beam degradation produce a strong background. Nevertheless, HRTEM pictures show that these spherical structures consist of a cluster of small crystalline nanoparticles between 5 and 15 nm in size (Figure 1c), which typically have a pseudo-hexagonal shape with pseudo-isoorientation. According to the Fast Fourier Transforms (FFT) of HRTEM images (Figure 1c *inset*), most of these crystals can be assigned to hematite (Fe_2_O_3_) with space group R-3c and lattice parameters of a = 5.038 Å, c = 13.772 Å. However, other phases have been detected (polyphasic), though it is not possible to unambiguously attribute their interplanar distances to a specific Fe-oxide phase. These rounded features are always closely associated with the crystalline nanospicules, and elemental analysis (EDX) reveals the presence of mainly Fe and O (Figure 1d). 

### 2.2. One Dimensional Gel Electrophoresis and Western Blotting Analysis of Ferritin in S. domuncula and Primmorph Tissues

**Figure 2:**
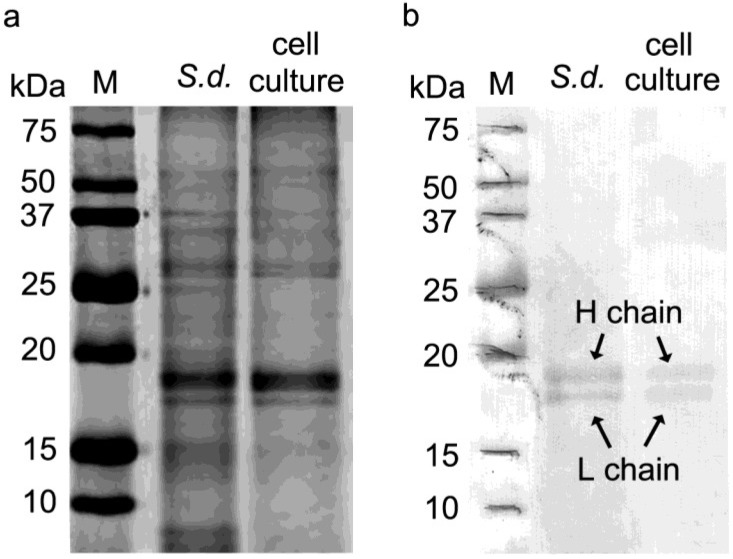
Gel electrophoresis and western blot for identification of ferritin in *S. domuncula* and primmorphs. *S. domuncula* and primmorph total extracts were prepared by homogenization in Low Salt Lysis Extraction Buffer (50 mM Tris pH 8.0 + 0.2 M NaCl). (**a**) Separation by one-dimensional 0.1% SDS–12% PAGE. The gel was stained with Coomassie Blue; (**b**) The proteins were transferred to a membrane and reacted with McAb-HF (Monoclonal Antibody to Human Ferritin IgG1, Cat. No. BM2161, Acris Antibodies GmbH, Hiddenhausen, Germany). Ferritin H and L chains from both sources display a molecular weight of 19 and 18 kDa, respectively. Size markers are given in kDa (lane M).

Tissue extracts from *S. domuncula* and primmorphs (10 days) were homogenized and loaded into a polyacrylamide gel (SDS-PAGE), and peptides were separated based on size (Figure 2a). Afterwards, the proteins were transferred onto a membrane and reacted with monoclonal Human Liver Ferritin IgG1 antibodies raised against human liver ferritin (McAb-HF). Figure 2b shows a positive cross-reaction of tissue extract with the anti-ferritin antibodies. Two bands are clearly visualized for *S. domuncula* and primmorph ferritin’s heavy chain (H) and light chain (L) at 19 and 18 kDa, respectively. The possibility that ferritin is found in *S. domuncula* and that primmorph tissue extract has a microbial origin was ruled out since microbial ferritin is composed of only one type of ferritin subunit [27]. 

Taken together, *S. domuncula*, as well as primmorphs, have ferritin in H and L chains with similar molecular weights when compared with other mammalian ferritins [28]. Moreover, the molecular weight found for *S. domuncula* ferritin heavy and light chains (H and L) matches the predicted theoretical molecular weights, 19.5 kDa for heavy chain (ferritin type 2, accession no. AJ306614) and 19.0 kDa for light chain (ferritin type 1, accession no. CAC84556) [29,30]. 

### 2.3. Sequence Alignment

**Figure 3:**
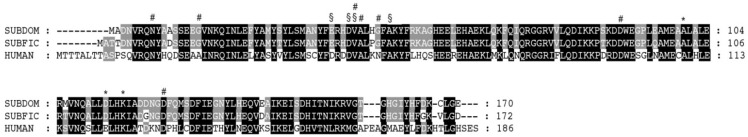
*S. domuncula* ferritin H chain (SUBDOM, accession number AJ306614.1), *S. ficus* H chain (SUBFIC, accession number AJ634779.1), and *Homo sapiens* H chain (accession number NM_002032.2) sequences were aligned. Residues conserved (similar or related with respect to their physico-chemical properties) in all sequences are shown in white on black and those in at least two sequences in black on gray. Typical ferritin region/amino acids were assigned. (*) Ferrihydrite nucleation center; (#) ferroxidase, and (§) ferrihydrite diiron center.

The H and L chains *of S. domuncula* ferritin mRNA were previously identified (Ferritin type 2, accession No. AJ306614 and ferritin type 1, accession no CAC84556) [30]. *S. domuncula* ferritin light chain (type 1) sequence is composed of 168 aa with an expected size of 19,011 kDa and a theoretical, slightly acidic pI of 5.31. The sequence for *S. domuncula* ferritin heavy chain (type 2) obtained is 170 aa long, and the calculated size is 19,535 kDa with a theoretical pI of 5.34. Highest sequence similarity (92%) was found for the ferritin heavy chain from *S. ficus* (type 2), with an expected *e*-value of 2 × 10^−88^ and 95% homology. Moreover, comparison between *Homo sapiens* heavy chain (Genbank accession no. AAI05803.1) and ferritin *S. domuncula* heavy chain shows 61% similarity and 73% homology with an expected *e*-value of 6 × 10^−51^ (Figure 3). This similarity agrees with results obtained by the western blot experiments. Identification of domains (iron ion channel, ferroxidase di-iron center and ferrihydrite nucleation center) were performed using NCBI database for conserved domains. The typical ferritin regions such as ferrihydrite nucleation center (Figure 3*), ferroxidase (Figure 3#), and ferrihydrite diiron center (Figure 3§) were found, and, moreover, an expected value of 1 × 10^−49^ was found when aligned with eukaryotic ferritin domains (Accession number cd01056, Euk_Ferritin, Eukaryotic Ferritin (Euk_Ferritin) [30].

### 2.4. Immunohistology of S. domuncula Filaments/Spicules and Tissue

**Figure 4:**
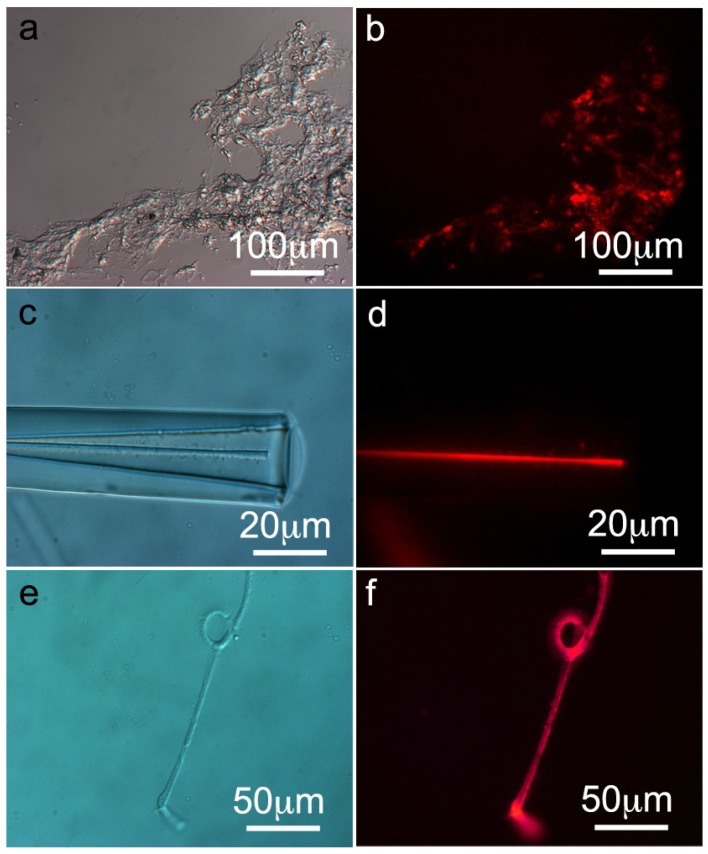
Presence and distribution of ferritin in tissue, spicule and axial filaments of *S. domuncula.* Thin cross-sections of *S. domuncula* tissue, spicules and filaments were treated with monoclonal Human Liver Ferritin IgG1 antibodies raised against human liver ferritin (McAb-HF, 1 h, RT). Afterwards, the samples were incubated with Cy3-label secondary anti-mouse antibodies, and the immunocomplexes were detected by fluorescence microscopy. (**a**) Light microscopic image of *S. domuncula* tissue cross-section; (**b**) Corresponding fluorescent image showing an intense fluorescent staining, indicating the presence of ferritin throughout the tissue; (**c**) Light microscopic image of HF slightly etched *S. domuncula* spicules; (**d**) Corresponding fluorescent image displaying a bright fluorescence signal at the axial filament; (**e**) Light microscopic image of *S. domuncula* axial filaments (**f**) Corresponding fluorescent image showing a strong cross-reaction with McAb-HF.

The presence and the distribution of ferritin in *S. domuncula* filaments, spicules and tissue were analyzed by immunochemistry techniques. Thin histological sections (8 μm thickness) of *S. domuncula* specimens were prepared as described in [7,11]. The thin sections were treated with monoclonal Human Liver Ferritin IgG1 antibodies raised against human liver ferritin (McAb-HF) for 1 h at RT. Afterwards, the histological sections were treated with Cy3-labeled secondary antibodies and the immunocomplexes were detected by fluorescence microscopy. At low magnification, tissue thin sections show a strong fluorescent red signal and wide distribution of ferritin throughout the sponge tissue cross-section (Figure 4a,b). As control, the primary antibody was replaced by blocking solution (PBS/BSA 0.3%, 1 h, RT), and no fluorescent signal was detected (Supplementary Information Figure S1). In addition, the same thin section were stained with Perls’ Prussian Blue stain for iron detection. The presence of iron oxide in *S. domucula* thin sections is observed (Supplementary Information Figure S2). These results are in agreement with STEM-HRTEM data (Figure 1). In addition, spicules (monoaxon) obtained from *S. domuncula* slightly etched with HF technique [7,8,11] were treated similarly. Figure 4d shows a clear signal indicating that ferritin is located within an/the axial filament. Subsequently, axial filaments from *S. domuncula* spicules were treated with McAb-HF displaying a strong fluorescent signal (Figure 4f). As controls, both filaments and spicules were treated with a solution of PBS/BSA 0.3% for 1 h at RT and analyzed under the fluorescence microscope. In this case, no signal was observed.

### 2.5. Elemental Analysis (ICP-MS) of S. domuncula Filaments

*S. domuncula* filaments and spicules were prepared as described elsewhere [31]. After the tissue degradation process, the spicules were obtained by sedimentation, and silica was dissolved with hydrofluoric acid (2 M HF/8 M NH_4_F, pH 5) and extensively washed with distilled water in order to remove inorganic ions. The filaments were obtained, treated accordingly (HNO_3_) and analyzed by ICP-MS. The results show the presence of elements such as Si, Al and Fe with the respective concentrations: 0.006 mg/L (Si) (0.122%), 0.001 mg/L (Al) (0.028%), and 0.003 mg/L (Fe) (0.076%), indicating the presence of iron very likely associated with ferritin, in agreement with previous findings (see Section 2.1 and Section 2.4). However, this assumption requires magnetic studies focusing on the axial filaments.

### 2.6. Magnetic Resonance Imaging (MRI) of S. domuncula Filaments and Spicules

A magnetic behavior has been attributed to ferritin polyphasic inorganic cores from different organisms [32,33]. The *S. domuncula* ferritin inorganic core shows the presence of hematite (Fe_2_O_3_), among other crystalline phases (polyphasic) (Section 2.1. Analysis of intracellular structures—Figure 1), and its magnetic behavior was analyzed by Magnetic Resonance Imaging (MRI).

**Figure 5:**
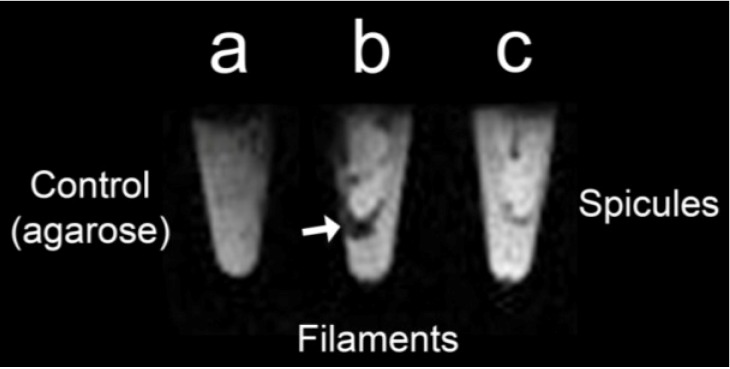
Magnetic Resonance Imaging (MRI) analysis of skeletal elements of *S. domuncula* immobilized into an agarose gel (1% w/v)*.* (**a**) Agarose gel (1% w/v) was used as the control, showing no signal; (**b**) axial filaments from *S. domuncula* show strong signals (dark spots) derived from T2*-weighted from the iron oxide agglomerates co-localized within the axial filaments (arrows); (**c**) Cleaned spicules do not show any signal/contrast.

*S. domuncula* axial filaments and spicules were immobilized in an agarose gel (1%w/v) (Figure 5). The agarose gel (Figure 5a) used as a control does not show any signal/contrast. On the other hand, Figure 5b shows the T2*-weighted image of immobilized *S. domuncula* axial filaments. The co-localization of axial filaments and the dark spots (MR image) indicate the presence of iron oxide nanoparticles inside the axial filaments, corroborating the data obtained from ICP-MS (Section 2.5. *Elemental analysis (ICP-MS) of S. domuncula filaments*) and demonstrating that the Demospongiae ferritin inorganic core displays excellent properties as a T1 contrast agent due to its magnetic properties. Nevertheless, cleaned *S. domuncula* spicules (no organics) analyzed in parallel do not show any signal, suggesting that magnetic iron oxide associated with ferritin polyphase is exclusively located in the axial filaments. It is known that iron is present in the spicule structure in trace quantities [34], but this is due to environmental contamination, and the iron species present does not display magnetic properties (Figure 5c).

## 3. Discussion

Sponges’s biomineralization process (spiculogenesis) in the animal model *S. domuncula* is well understood [7,11]. The microscopy analysis (STEM-HRTEM) of ultrathin cuts of primmorphs shows agglomerate spherical features, with a diameter of approximately 50 nm and always in close association with the nanosized crystalline rods, located inside the sclerocytes [16]. A detailed analysis of these structures revealed pseudo-hexagonal nanostructures with a size varying between 5 and 15 nm and composed essentially of elements such as Fe and O displaying a pseudo-orientation. Although most of these crystals belong to the hematite (Fe_2_O_3_) phase, unattributed phases have been detected (polyphasic), suggesting the presence of ferritin cores. The crystallographic structure of the ferritin inorganic core has remained a difficult problem to solve. In the early 60s, ferritin’s respective cores were analyzed by X-ray diffraction (XRD) and electron diffraction (ED), revealing an amorphous and paracrystalline-to-crystalline mixture. Thus, it was named according to its similarity with its natural counterpart, ferrihydrite [21,22].

The best accepted model for the ferritin inorganic core is the presence of at least 3 polymorphs/phases, which differ in ratio depending on the organism [35]. Ferritin cores of horse spleen analyzed by both HRTEM and ED displays a polyphasic structure [36,37]. Concerning the sponges, the only report on the presence of iron oxides particles (granules) was published by Towe *et al.* in the late 60s and stated that an iron oxide polymorph, lepidocrocite (γ-FeOOH), was embedded in the spongin fibres of a keratose sponge (extracellularly) [21].

Polyphasic iron oxide, among them Fe_2_O_3_, found within sclerocytes resembles the inorganic core typical of ferritin. Consequently, it prompted us to analyze the presence of ferritin within *S. domuncula* and in respective primmorphs. However, the presence of ferritin as not an expected finding. It is widely known that biological systems developed mechanisms to accommodate iron under the several possible iron oxide polymorphs where iron is sequestered and rendered inert [5]. A co-relation between ferritin and spiculogenesis (e.g., silicatein) was proposed earlier, but only at the gene expression level [30]. It was equally demonstrated that when the growth medium (seawater) was supplemented with Fe(III) (30 μM) in addition to the silicate source (60 μM) (optimal concentrations), primmorphs grew 6–12 mm longer than regular primmorphs. Both silicatein and ferritin genes were strongly upregulated [30,38]. Based on these findings, it was proposed that these elements (silicate and iron) are involved in spiculogenesis and morphogenesis by stimulating the expression of silicatein and ferritin [30,38].

According to an evolutionary adaptation perspective, silica-biomineralizing organisms, such as diatoms and sponges, have developed mechanisms to overcome low silica availability in seawater by depositing large amounts of amorphous silica within their bodies in silica reservoirs exclusively present in sclerocytes. For example, it was recently demonstrated that siliceous sponges form amorphous, silica-containing vesicles termed silicasome. An underlying accumulation mechanism in other biomineralizing organisms, marine sponges use these silica-filled internal vesicles (silicasomes) to create supersaturated conditions in defined intracellular spaces so that a controlled biosilification can occur [15]. 

**Figure 6:**
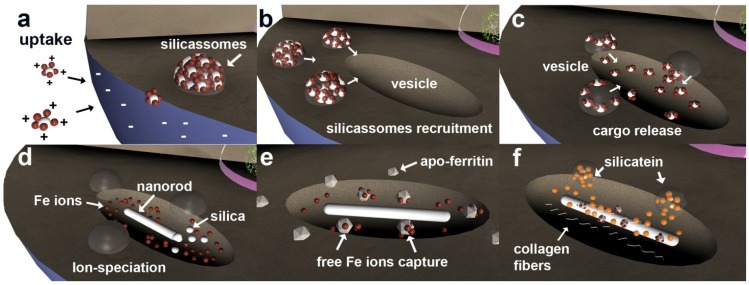
Schematic representation of early stages of spiculogenesis development involving ferritin. (**a**) Positively charged Si/Fe complexes are formed extracellularly. The difference in charges allows an efficient uptake mechanism that leads to the formation of silicasome; (**b**) Silicasome recruitment upon cellular triggering of spiculogenesis; (**c**) Silicasome release their *cargo* into the specialized intracellular vesicles; (**d**) Ion-speciation mechanism. The crystalline nanorods are synthesized using Al and Si elements, and free iron ions are released; (**e**) Free iron ions are captured by apo-ferritin (agglomerates 50 nm) lowering their toxicity and preventing ionic diffusion and, ultimately, cellular collapse; (**f**) Adhesion of ferritin onto the crystalline nanorod and integration into the axial filament together with collagen and silicatein.

However, under normal conditions (open ocean) silica ions (SiO_4_ or SiO_3_^−^) are not freely available. These negatively charged molecules are always associated in nature with positively charged iron ions [39,40,41,42]. In primmorphs 3D culture, the iron-silica complexes are formed, creating positively charged, shielded silica molecules that interact with sponge cell surfaces, leading to more efficient silica uptake and formation of intracellular vesicles—silicasomes (Figure 6a). These supersaturated silica-iron compartments create an advantageous intracellular environment used by the sponge cells for initializing spicule formation. This is in agreement with former observations, using *S. domuncula* primmorphs, that silicatein gene expression is upregulated in the presence of silica (60 μM), with a dramatic increase in gene expression in the presence of both iron and silica components [17,30,38,43]. 

From this perspective, silicasomes are used by sponge cells for initializing spicule formation, *i.e.*, silicasomes are recruited close to intracellular vesicles (Figure 6b) where their cargo is released (Figure 6c). Afterwards, an ion-speciation mechanism occurs (Figure 6d), where silica and aluminium elements form the crystal nanostructure (nanorod) that acts as a seed for further spicule growth (also energetically favorable) [16]. The free and unused ions (known to be highly toxic due to their ROS production ability) are captured by apo-ferritin cages and oxidized by their ferroxidase site, rendering the iron ions inert (Figure 6e) [44].

These findings suggest that iron ions contribute indirectly to spiculogenesis, *i.e.*, are initially used by the sponge cells for efficient silica uptake and silicasome formation. The presence of iron (oxide) inside *S. domuncula* spicule axial filaments (but not spicules), as confirmed by means of ICP-MS analyses, immunostaining techniques and Magnetic Resonance Imaging (MRI), was an unexpected finding. Of interest is the fact that zeolites (aluminium-silicates) are commonly used in protein purification as an alternative or complement to several biochemical methods [45,46,47] due to their great affinity for proteins, such as ferritin [48]. By analogy with zeolite chemistry and its interaction with proteins, the intracelullar crystalline nanorod can also adsorb proteins such as silicatein and/or ferritin onto its surface (Figure 6f), creating a templated protein deposition with an atomic precision [16,49,50,51,52]. At this very early stage of spiculogenesis, the nanorods further adsorbed silicatein and ferritin embedded within a collagen matrix through a continuous process.

In the last step, the spicule is extruded to the extracellular space where it grows in length and thickness [7,8,11]. Consequently, it can be suggested that the excess iron (oxidized and localized within ferritin rings) is being integrated into the axial filament in a mechanism of cellular purge and regeneration.

## 4. Experimental Section

### 4.1. Primmorphs Cell Culture

Living specimens of *Suberites domuncula* (Porifera, Demospongiae, Hadromerida) had been collected in the Adriatic Sea near Rovinj (Croatia) and kept in aquaria at a temperature of 17 °C prior to their use. Single cells were obtained as described elsewhere [12,13]. Briefly, tissue samples were cut into cubes and single cells were obtained by dissociation with Ca^2+^- and Mg^2+^-free artificial seawater (CMFASW), containing 2.5 mM EDTA. The cell suspension was centrifuged, and the final pellet was re-suspended in CMFASW, supplemented with 0.1% of RPMI 1640 medium (Sigma-Aldrich, Taufkirchen, Germany) and 60 μM silicic acid (as sodium metasilicate; Sigma-Aldrich, Taufkirchen, Germany). Primmorphs of at least 1 mm in diameter were formed after 5 days and used for further experiments.

### 4.2. Sample Preparation and Microscopy Analysis

Primmorph samples were cut into pieces (2 mm^3^), incubated in 0.1 M phosphate buffer (supplemented with 2.5% glutaraldehyde, 0.82% NaCl [pH 7.4]), and then washed in 0.1 M phosphate buffer (1.75% NaCl) at room temperature. After the samples had been treated with 1.25% NaHCO_3_, 2% OsO_4_, and 1% NaCl, they were dehydrated with ethanol. The dried samples were incubated with propylene oxide, fixed in propylene oxide/Araldite (2:1), covered with pure Araldite, and hardened at 60 °C for 2 days prior to being cut into 60-nm ultrathin slices (Ultracut S; Leica, Wetzlar, Germany).

The samples were finally transferred onto coated copper grids. TEM analysis was carried out with a FEI Tecnai F30 S-TWIN transmission electron microscope equipped with field emission gun and scanning unit. High-resolution images (HRTEM) were taken with a CCD camera (14-bit GATAN 794MSC) and acquired by Gatan DigitalMicrograph software (Gatan GmbH, München, Germany). STEM was performed using the Tecnai Microprobe STEM mode (FEI Munich, Gräfelfing, Germany) with a slightly defocused beam in order to reduce the damage to the organic tissue. STEM images were collected by a Fischione high angular annular dark field detector (HAADF, FEI Munich, Gräfelfing, Germany). Energy dispersive X-ray analysis (EDX) was undertaken in STEM mode and quantified using FEI ESVision software (FEI Munich, Gräfelfing, Germany).

### 4.3. Protein Extraction

For protein extraction, *S. domuncula* and primmorphs were centrifuged at 1000× rpm for 5 min at +4 °C to remove the culture medium and then resuspended in 1 mL of low salt lysis buffer (50 mM Tris-HCl, pH 7.5; 1% Triton X-100), containing 10 μL of protease inhibitor cocktail. The samples were sonicated for 10 s followed by centrifugation at 12,000*×* rpm for 10 min at +4 °C to pellet the cell debris. The supernatant was collected, and the protein content was determined following the Bradford method [53].

### 4.4. One-Dimensional Gel Electrophoresis and Western Immunoblotting

Extracts (4 μg per lane) were subjected to electrophoresis on a 12% polyacrylamide gel according to Laemmli [54]. Protein samples were denatured in loading buffer containing β-mercaptoethanol at 95 °C for 5 min. The run was carried out in Mini-PROTEAN 3 electrophoresis system, using a constant current of 120 V for 90 min with Tris-Glycerin-SDS (TGS) running buffer. Staining of proteins was performed with Coomassie Blue (Pierce, Thermo Fisher Scientific BV & Co KG, Bonn, Germany). Polypeptides were then transferred to a polyvinylidene fluoride (PVDF) membrane. The run was carried out by applying 0.06 mA/cm^2^ in a Trans-Blot SD semi-dry transfer cell (BIO-RAD, München, Germany) for 1 h. The membrane was kept at +4 °C overnight in blocking solution containing PBS/BSA [bovine serum albumin] (3%). The first antibody applied to the membrane was the Monoclonal Antibody to Human Liver Ferritin IgG1 produced in mouse (McAb-HF) (Cat. No. BM2161, Acris Antibodies GmbH, Herford, Germany) (1:500 dilution in PBS/BSA) incubated for 1 h at room temperature. The blots were incubated with the second antibody, anti-rabbit IgG Alkaline Phosphatase (1:2000 dilution), for 1 h at room temperature. The immunocomplexes were visualized with the color develop system, NBT/BCIP-system (Sigma-Aldrich, Taufkirchen, Germany).

### 4.5. Sequence Analysis of Ferritin Deduced Protein

The sequences were analyzed with computer programs Blast [55] and FASTA [56]. Multiple alignments were performed with CLUSTAL W Ver. 2 [57]. 

### 4.6. Preparation of Suberites Domuncula Spicules and Axial Filaments

*Suberites domuncula* filaments and spicules were prepared as described elsewhere [31]. Briefly, tissue samples were treated first with sulfuric acid/nitric acid and then with *n*-butanol/water/SDS. The spicules were than collected by sedimentation. The axial filaments were isolated from the spicules by dissolution of the silica material with hydrofluoric acid (2 M HF/8 M NH_4_F, pH 5) at room temperature overnight and were then collected, dialyzed against distilled water, collected by centrifugation and air-dried [6].

### 4.7. Histological Preparation of Suberites Domuncula Tissue

*Suberites domuncula* tissue were fixed with paraformaldehyde (4% in sea water; Sigma) for 30 min and then washed with PBS at room temperature and embedded in Tissue-Tek (Science Service, München, Germany). 8-μm-thick frozen sections were obtained using a microtome as previously described [7,11].

### 4.8. Immunohistology of Suberites Domuncula Filaments, Spicules and Tissue

For immunohistological analysis of frozen *S. domuncula* tissue cross-sections, the 8-μm-thick slices were blocked with 0.3% bovine serum albumin (BSA) (Carl Roth GmbH + Co. KG, Karlsruhe, Germany) in Phosphate Buffered Saline pH 7.4 (PBS) (AppliChem, Darmstadt, Germany) overnight at +4 °C and washed with TBS-T buffer (Tris-HCl pH 8.0; 150 mM NaCl, 0.05% Tween^®^-20) (Sigma-Aldrich, Taufkirchen, Germany). Glass slides containing frozen *S. domuncula* tissue cross-sections, filaments and spicules were incubated with the primary Human Liver Ferritin IgG1 produced in mice (McAb-HF) (Cat. No. BM2161, Acris Antibodies GmbH, Herford, Germany) (1:1000) in PBS/BSA 3% for 1 h at room temperature. Afterwards, glass slides containing frozen *S. domuncula* tissue cross-sections, filaments and spicules were washed once more for 3 times with TBS-T for 5 minutes and incubated with secondary antibody, Cy™-3 conjugated Goat Anti-Mouse IgG (H + L) (1:1000) (Code 111-165-045, Lot 37329, Jackson Immune Research Inc., Suffolk, UK) prepared in PBS/BSA 0.3% for 1 h at room temperature. As control, the first antibody was replaced by blocking solution (PBS/BSA 0.3%) for 1 h at room temperature. The fluorescence analysis was performed with an Olympus AHBT3 light microscope, together with an AH3-RFC reflected light fluorescence attachment at the emission wavelength of 546 nm (filter G).

*S. domuncula* tissue frozen cross-sections were treated with Perls’ Prussian blue staining solution. Immediately before using, 20% hydrochloric acid (35%–38% HCl, Sigma-Aldrich) and 10% potassium ferrocyanide (KFe(CN)_6_, Sigma-Aldrich, Taufkirchen, Germany) were mixed in equal parts. Afterwards, the thin cross-sections were immersed in freshly prepared Perls’ blue staining solution for 40 min at room temperature. The samples were then washed three times with distilled water and observed under light microscope (Olympus AHBT3 light microscope, Hamburg, Germany).

### 4.9. Induced Couple Plasma—Mass Spectrometry (ICP-MS) Analysis of S. domuncula Filaments

Filaments (5 mg) were treated with 2% concentrated nitric acid (HNO_3_, Acros Organic) for 30 min at 110 °C. The solution was left to cool down until it reached room temperature, and the sample was then loaded into an ICP-MS spectrometer (Thermo Element II, Agilent 4500, VG Elemental Plasma Quad3, Agilent Technologies, Böblingen, Germany) for elemental analysis.

### 4.10. MRI Measurements, Imaging and Computer Tomography (CT)

Magnetic resonance imaging (MRI) was performed on a clinical 3.0 Tesla whole body scanner (Magnetom Trio, Siemens Medical Solutions, Erlangen, Germany). The *S. domuncula* filaments, and spicules were immobilized into an agarose gel (1% w/v). As control, agarose gel (1% w/v) was used in parallel. For radio frequency excitation and signal reception, an 8-channel knee coil (Siemens Medical Solutions, Erlangen, Germany) was used. T1-weighted imaging was performed using a 2D FLASH (fast low angle shot) pulse sequence with the following sequence parameters: repetition time (TR) = 350 ms, echo time (TE) = 3.5 ms, flip angle (FA) = 70°. For proton density and T2-weighted imaging, a CPMG (Carr-Purcell-Meighboom-Gill) pulse sequence (TR = 7780 ms, FA = 90°) with 21 different echo times from 8.5 ms to 178.5 ms was used. T2*-weighting was performed using a 2D FLASH pulse sequence with TR = 620 ms, TE = 20 ms, FA = 20°. The voxel size in all cases was 0.3 × 0.3 × 3.0 mm^3^.

## 5. Conclusions

Iron has a paradoxical role in biology. It is a fundamental element due to its involvement in many cellular/biochemical pathways essential for life, but it also has the ability to form reactive oxygen species (ROS) that interact with metabolic machinery, leading ultimately to cell death. Biomineralization of iron was developed by a wide range of evolutionarily distinct organisms to render iron inert (ferritin) or increase strength in skeletal elements (e.g., chiton’s teeth). However, a dual role played by the same biomineralization process has never been attributed to a biological system. The *S. domuncula* biomineralization model is best understood. Here the presence of iron in the growing medium has been known to upregulate silicatein and ferritin gene expression. We have demonstrated the presence of ferritin and its inorganic core within sponge specialized cells (sclerocytes). Ferritin acts as a scavenger for free labile iron and simultaneously enables efficient silica uptake.

In the early stages of spiculogenesis, ferritin is further integrated through an adsorption process. This is corroborated by the presence of ferritin exclusively localized within the axial filaments of mature spicules of *S. domuncula*. We propose that sponges undergo highly efficient biomineralization processes of iron and silica, making use of both Fe and Si for uptake and further using an ion-speciation associated mechanism.

Taking nature as a model, but using advanced molecular biology techniques to express apo-ferritin, the environmental depletion of sponges can be avoided, and the controlled/templated synthesis of nanoparticles with different chemical natures [58], which find a wide range of applications in the medical field and magnetic resonance imaging, can be achieved.

## Supplementary Files

Supplementary File 1

## References

[1] Xiao S., Yuan X., Knoll A.H. (2000). Eumetazoan fossils in terminal Proterozoic phosphorites?. Proc. Natl. Acad. Sci. USA.

[2] Müller W.E.G., Li J., Schröder H.C., Qiao L., Wang X.H. (2007). The unique skeleton of siliceous sponges (Porifera; Hexactinellida and Demospongiae) that evolved first from the Urmetazoa during the Proterozoic: A review. Biogeosciences.

[3] Knoll A.H., Carroll S.B. (1999). Early animal evolution: Emerging views from comparative biology and geology. Science.

[4] Walker G. (2003). Snowball Earth: The Story of the Great Global Catastrophe that Spawned Life as We Know It.

[5] Lowenstam H.A., Weiner S. (1989). On Biomineralization.

[6] Cha J.N., Shimizu K., Zhou Y., Christianssen S.C., Chmelka B.F., Stucky G.D., Morse D.E. (1999). Silicatein filaments and subunits from a marine sponge direct the polymerization of silica and silicones *in vitro*. Proc. Natl. Acad. Sci. USA.

[7] Müller W.E.G., Rothenberger M., Boreiko A., Tremel W., Reiber A., Schröder H.C. (2005). Formation of siliceous spicules in the marine demosponge *Suberites domuncula*. Cell Tissue Res..

[8] Müller W.E.G., Wang X.H., Belikov S.I., Tremel W., Schloßmacher U., Natoli A., Brandt D., Boreiko A., Tahir M.N., Müller I.M., Bäuerlein E. (2007). Formation of siliceous spicules in demosponges: Example *Suberites domuncula*. Handbook of Biomineralization Volume 1, The Biology of Biominerals Structure Formation.

[9] Müller W.E.G., Jochum K., Stoll B., Wang X.H. (2008). Formation of giant spicule from quartz glass by the deep sea sponge *Monorhaphis*. Chem. Mater..

[10] Wang X.H., Boreiko A., Schloßmacher U., Brandt D., Schröder H.C., Li J., Kaandorp J.A., Götz H., Duschner H., Müller W.E.G. (2008). Axial growth of hexactinellid spicules: Formation of cone-like structural units in the giant basal spicules of the hexactinellid *Monorhaphis*. J. Struct. Biol..

[11] Müller W.E.G., Belikov S., Tremel W., Perry C.C., Gieskes W.W.C., Boreiko A., Schröder H.C. (2006). Siliceous spicules in marine demosponges (example *Suberites domuncula*). Micron.

[12] Custodio R.M., Prokic I., Steffen R., Koziol C., Borojevic R., Brümmer F., Nickel M., Müller W.E.G. (1998). Primmorphs generated from dissociated cells of the sponge *Suberites domuncula*: A model system for studies of cell proliferation and cell death. Mech. Ageing Dev..

[13] Müller W.E.G., Wiens M., Batel R., Steffen R., Borojevic R., Custodio R.M. (1999). Establishment of a primary cell culture from a sponge: Primmorphs from *Suberites domuncula*. Mar. Ecol. Progr. Ser..

[14] Schröder H.C., Perovic-Ottstadt S., Rothenberger M., Wiens M., Schwertner H., Batel R., Korzhev M., Müller I.M., Müller W.E.G. (2004). Silica transport in the demosponge *Suberites domuncula*: Fluorescence emission analysis using the PDMPO probe and cloning of a potential transporter. Biochem. J..

[15] Schröder H.C., Natalio F., Shukoor M.I., Tremel W., Schloßmacher U., Wang X., Müller W.E.G. (2007). Apposition of silica lamellae during growth of spicules in the demosponge *Suberites domuncula*: Biological/biochemical studies and chemical/biomimetical confirmation. J. Struct. Biol..

[16] Mugnaioli E., Natalio F., Schloβmacher U., Wang X., Müller W.E.G., Kolb U. (2009). Crystalline nanorods as possible templates for the synthesis of amorphous biosilica during spicule formation in Demospongiae. ChemBioChem.

[17] Krasko A., Batel R., Schröder H.C., Müller I.M., Müller W.E.G. (2000). Expression of silicatein and collagen genes in the marine sponge *Suberites domuncula* is controlled by silicate and myotrophin. Eur. J. Biochem..

[18] Jacobs A., Worwood M. (1980). Editors: Iron in Biochemistry and Medicine II.

[19] Blakemore R. (1975). Magnetotactic Bacteria. Science.

[20] Kirschvink J.L., Jones D.S., MacFadden B.J. (1985). Magnetite Biomineralization and Magnetoreception in Organisms: A New Biomagnetism (Topics in Geobiology).

[21] Towe K.M., Rützler K. (1968). Lepidocrocite Iron Mineralization in Keratose Sponge Granules. Science.

[22] Towe K.M., Bradley W.P. (1967). Mineralogical constitution of colloidal hydrous ferric oxides. J. Colloid Interface Sci..

[23] Mann S., Bannister J.V., Williams R.J.P. (1986). Structure and composition of ferritin cores isolated from human spleen, limpet (*Patella vulgata*) hemolymph and bacterial (*Pseudomonas aeruginosa*) cells. J. Mol. Biol..

[24] Massover W.H., Cowley J.M (1973). The ultrastructure of ferritin macromolecules. The lattice structure of the core crystallites. Proc. Nat. Acad. Sci. USA.

[25] Mayer D.E., Rohrer J.S., Schoeller D.A., Harris D.C. (1983). Fate of oxygen during ferritin iron incorporation. Biochemistry.

[26] Allen M.A., Prissel M.M., Young M.J., Douglas T., Bäuerlein E. (2007). Constrained metal oxide mineralization: Lessons from ferritin applied to other protein cage architectures. Handbook of Biomineralization Volume 1, Biomimetic and Bioinspired Chemistry.

[27] Theil E.C., Hase T. (1993). Plant and microbial ferritins. Iron Chelation in Plants and Soil Microorganisms.

[28] Theil E.C. (1987). Ferritin: structure, gene regulation, and cellular function in animals, plants, and microorganisms. Annu. Rev. Biochem..

[29] Artimo P., Jonnalagedda M., Arnold K., Baratin D., Csardi G., de Castro E., Duvaud S., Flegel V., Fortier A., Gasteiger E. (2012). ExPASy: SIB bioinformatics resource portal. Nucleic Acids Res..

[30] Krasko A., Schroder H.C., Batel R., Grebenjuk V.A., Steffen R., Muller I.M., Muller W.E.G. (2002). Iron induces proliferation and morphogenesis in primmorphs from the marine sponge *Suberites domuncula*. DNA Cell Biol..

[31] Kaluzhnaya O.V., Belikov S.I., Schröder H.C., Rothenberger M., Zapf S., Kaandorp J.A., Borejko A., Müller I.M., Müller W.E.G. (2005). Dynamics of skeletal formation in the Lake Baikal sponge *Lubomirskia baicalensis*. Part I: Biological and biochemical studies. Naturwissenschaften.

[32] Brooks R.A., Vymazal J., Goldfarb R.B., Bulte J.W., Aisen P. (1998). Relaxometry and magnetometry of ferritin. Magn. Reson. Med..

[33] Meldrum F.C., Heywood B.R., Mann S. (1992). Magneto-ferritin: *In vitro* synthesis of a novel magnetic protein. Science.

[34] Sandford F. (2003). Physical and chemical analysis of the siliceous skeletons in six sponges of two groups (demospongiae and hexactinellida). Microsc. Res. Tech..

[35] Cornell R.M., Schwertmann U. (2003). The Iron Oxides.

[36] Cowley J.M., Janney D., Gerkin R.C., Buseck P.R. (2000). The structure of ferritin cores determined by electron nanodiffraction. J. Struct. Biol..

[37] Quintana C., Bonnet N., Jeantet A.Y., Chemel P. (1987). Crystallographic study of the ferritin molecule: New results obtained from natural cystals *in situ* (mollusc oocyte) and from isolated molecules horse spleen. Biol. Cell.

[38] Müller W.E.G., Krasko A., Le Pennec G., Schröder H.C. (2003). Biochemistry and Cell Biology of Silica Formation in Sponges. Microsc. Res. Tech..

[39] Jambor J.L., Dutrizac J.E. (1998). Occurrence and Constitution of Natural and Synthetic Ferrihydrite, a widespread Iron Oxyhydroxide. Chem. Rev..

[40] Parfitt R.L., Vandergaast S.J., Childs C.W. (1992). A structural model for natural siliceous ferrihydrite. Clays Clay Miner..

[41] Davis C.C., Chen H.-W., Edwards M. (2002). Modeling silica sorption to iron hydroxide. Environ. Sci. Technol..

[42] Hydes D.J. (1979). Aluminum in Seawater: Control by Inorganic Processes. Science.

[43] Le Pennec G., Perovic S., Ammar S.M.A., Grebenjuk V.A., Steffen R., Brümmer F., Müller W.E.G. (2003). Cultivation of primmorphs from the marine sponge *Suberites domuncula*: Morphogenetic potential of silicon and iron. A review. J. Biotechnol..

[44] Chasteen N.D., Harrison P.M. (1999). Mineralization in ferritin: An efficient means of iron storage. J. Struct. Biol..

[45] Loader C.E., Timmons C.J. (1967). Studies in photochemistry. Part V. The photocyclodehydrogenation of 2-furyl- and 2-thienyl-ethylenes: The mass spectra of the products. J. Chem. Soc. C.

[46] Antelo B., Castedo L., Delamano J., Gómez A., López C., Toto G. (1996). Photochemical Ring Closure of 1-Tosyl-1,2-diarylethenes. J. Org. Chem..

[47] Karmininski-Zamola G., Fiser-Jakic L., Jakopcic K. (1982). Photochemistry of furans. Photochemical transformations of some substituted 2-phenyl-3-furylacrylic acids. Tetrahedron.

[48] Klint D., Karlson G., Bovin J.-O. (1990). Cryo-TEM Snapshots of ferritin adsorbed on small zeolite crystals. Angew. Chem. Int. Ed..

[49] Imsiecke G., Müller W.E.G. (1995). Unusual presence and intranuclear storage of silica crystals in the freshwater sponges *Ephydatia muelleri* and *Spongilla lacustris* (Porifera: Spongillidae). Cell. Mol. Biol..

[50] Croce G., Frache A., Milanesio M., Viterbo D., Bavestrello G., Benatti U., Giovine M., Amenitsch H. (2003). Fiber diffraction study of spicules from marine sponges. Microsc. Res. Tech..

[51] Garrone R. (1969). Collagène, spongine et squelette minéral chez l'éponge Haliclona rosea (O.S.). J. Micros..

[52] Donadey C., Paris J., Vacelet J., Rützler K. (1990). Occurrence and ultrastructure of microraphides in *Axinella polypoides*. New Perspectives in Sponge Biology (International Conference on the Biology of Sponges, 1985).

[53] Bradford M.M. (1976). A rapid and sensitive method for the quantitation of microgram quantities of protein utilizing the principle of protein-dye binding. Anal. Biochem..

[54] Laemmli U.K. (1970). Cleavage of structural proteins during the assembly of the head of bacteriophage T4. Nature.

[55] Altschul S.F., Gish W., Miller W., Myers E.W., Lipman D.J. (1990). Basic local alignment search tool. J. Mol. Biol..

[56] Pearson W.R., Lipman D.J. (1988). Improved tools for biological sequence comparison. Proc. Natl. Acad. Sci. USA.

[57] Larkin M.A., Blackshields G., Brown N.P., Chenna R., McGettigan P.A., McWilliam H., Valentin F., Wallace I.M., Wilm A., Lopez R. (2007). ClustalW and ClustalX version 2. Bioinformatics.

[58] Dickerson M.B., Naik R.R., Sarosi P.M., Agarwal G., Stone M.O., Sandhage K.H. (2005). Ceramic Nanoparticle Assemblies with Tailored Shapes and Tailored Chemistries via Biosculpting and Shape-Preserving Inorganic Conversion. J. Nanosci. Nanotech..

