# Supplementary material for: Localization and Characterization of Ferritin in Demospongiae: A Possible Role on Spiculogenesis
Source: Mar Drugs. 2014 Aug 22;12(8):4659–76. doi: 10.3390/md12084659 (PMC4145336; doi:10.3390/md12084659)
Supplement: Supplementary File 1 [file marinedrugs-12-04659-s001.pdf]

Supplementary Information

Figure S1. Control experiments of immunostaining of *S. domuncula* tissue cross-sections. Sections were treated with blocking solution (PBS/BSA 0.3%, 1 h RT) and Cy3-labelled secondary antibody (1 h, RT). (**a**) Light microscope image and (**b**) corresponding fluorescent image showing no signal. Scale bar: 100 µm.

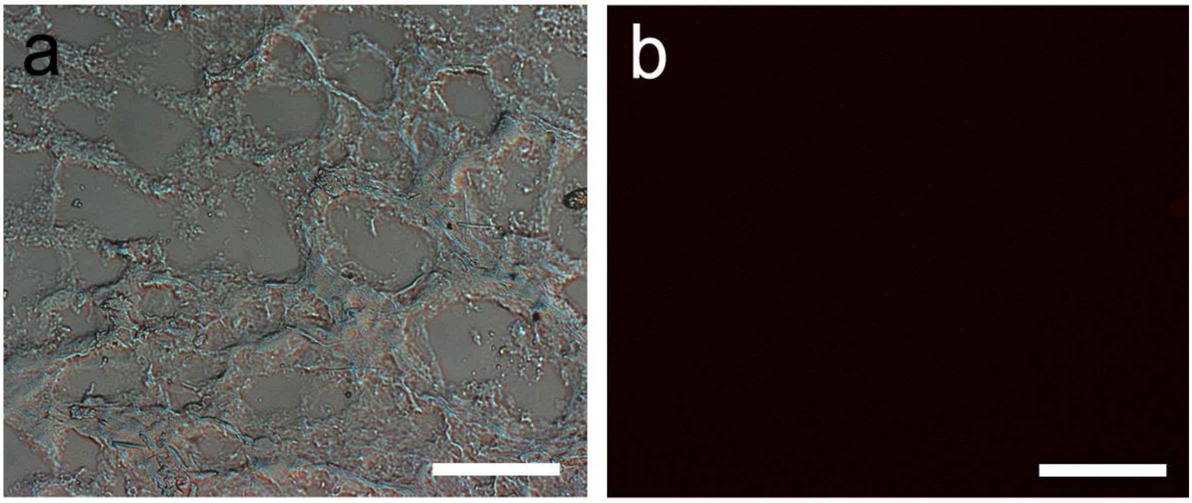

Figure S2. Light microscope images of *S. domuncula* tissue cross-sections after staining with Perls' Prussian Blue solution. Small spheres dispersed throughout tissue cross-sections are clearly observed.

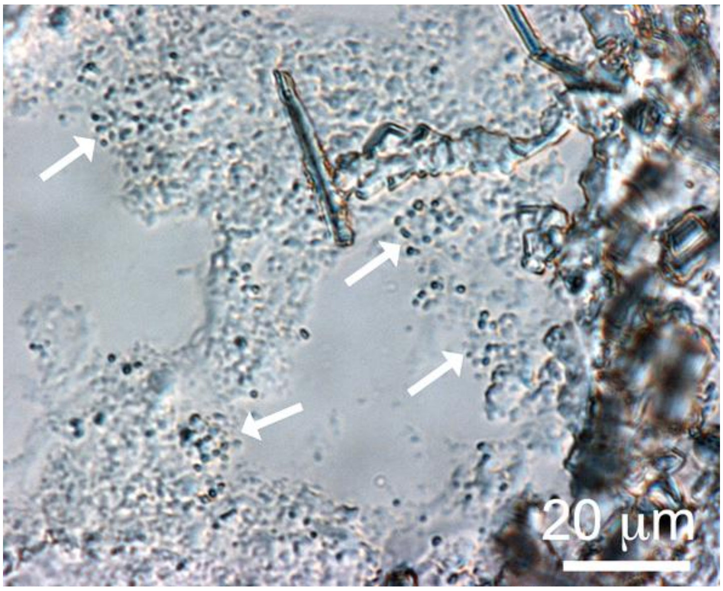

© 2014 by the authors; licensee MDPI, Basel, Switzerland. This article is an open access article distributed under the terms and conditions of the Creative Commons Attribution license (http://creativecommons.org/licenses/by/3.0/).
